# Heterogeneous nuclear ribonucleoprotein K is over expressed, aberrantly localised and is associated with poor prognosis in colorectal cancer

**DOI:** 10.1038/sj.bjc.6603349

**Published:** 2006-09-05

**Authors:** B Carpenter, M McKay, S R Dundas, L C Lawrie, C Telfer, G I Murray

**Affiliations:** 1 Department of Pathology, University of Aberdeen, Foresterhill, Aberdeen AB25 2ZD, UK; 2 Auvation Ltd, Crombie Lodge, Aberdeen Science Park, Balgownie Drive, Aberdeen, UK

**Keywords:** colorectal cancer, hnRNP K, immunohistochemistry, monoclonal antibody proteomics

## Abstract

Heterogeneous ribonucleoprotein K (hnRNP K) is a member of the hnRNP family which has several different cellular roles including transcription, mRNA shuttling, RNA editing and translation. Several reports implicate hnRNP K having a role in tumorigenesis, for instance hnRNP K increases transcription of the oncogene c-myc and hnRNP K expression is regulated by the p53/MDM 2 pathway. In this study comparing normal colon to colorectal cancer by proteomics, hnRNP K was identified as being overexpressed in this type of cancer. Immunohistochemistry with a monoclonal antibody to hnRNP K (which we developed) on colorectal cancer tissue microarray, confirmed that hnRNP K was overexpressed in colorectal cancer (*P*<0.001) and also showed that hnRNP K had an aberrant subcellular localisation in cancer cells. In normal colon hnRNP K was exclusively nuclear whereas in colorectal cancer the protein localised both in the cytoplasm and the nucleus. There were significant increases in both nuclear (*P*=0.007) and cytoplasmic (*P*=0.001) expression of hnRNP K in Dukes C tumours compared with early stage tumours. In Dukes C patient's good survival was associated with increased hnRNP K nuclear expression (*P*=0.0093). To elaborate on the recent observation that hnRNP K is regulated by p53, the expression profiles of these two proteins were also analysed. There was no correlation between hnRNP K and p53 expression, however, patients who presented tumours that were positive for hnRNP K and p53 had a poorer survival outcome (*P*=0.045).

Initial reports describe the heterogeneous ribonucleoprotein (hnRNP) family as a group of six proteins which bound RNA II polymerase transcripts to form hnRNP particles. Subsequently immuno-purification of the hnRNP complex identified additional factors (Choi and Dreyfuss, 1984); there are in excess of 20 hnRNPs and interestingly, certain family members are emerging as having important roles in tumour development (Carpenter *et al*, 2006). Heterogeneous ribonucleoprotein A2/B1 is the most extensively studied member which is overexpressed in different tumour types and preliminary studies indicate hnRNP A2/B1 may be a good prognostic and diagnostic marker (Carpenter *et al*, 2006). Heterogeneous ribonucleoprotein K is another hnRNP family member that has been implicated in regulating tumorigenesis.

Heterogeneous ribonucleoprotein K is a 464 amino-acid protein with 3K homology domains (KH) that mediate DNA and RNA binding (Bomsztyk *et al*, 2004) and contains both nuclear localisation and nuclear shuttling domains (Bomsztyk *et al*, 1997). Based on these domains it was presumed that the hnRNP K protein is involved in multiple steps of gene expression including transcription, RNA splicing and translation; such presumptions were later confirmed. Heterogeneous ribonucleoprotein K was shown to bind DNA in a CT element-dependent manner (Takimoto *et al*, 1993). Of interest, such an element is present in the oncogene c-myc, and overexpression of hnRNP K increases the transcriptional activity of a c-myc gene reporter (Takimoto *et al*, 1993). Also hnRNP K in combination with the transcription factor SP1, binds and activates the c-src promoter (Ritchie *et al*, 2003). Heterogeneous ribonucleoprotein K involvement in splicing was demonstrated using the chicken *β* tropomyosin gene, whose alternative splicing of exon 6A is mediated by a downstream intronic enhancer. Heterogeneous ribonucleoprotein K was identified as a protein present in the intronic enhancer complex whose function is to activate splicing of exon 6A (Expert-Bezancon *et al*, 2002). Heterogeneous ribonucleoprotein K also mediates translation, in HeLa cells hnRNP K silences translation of a luciferase reporter mRNA containing the differentiation control element present in the 3′UTR of the 15 lipoxygenase gene (Ostareck-Lederer *et al*, 1994). Furthermore, hnRNP K stimulates translation of a c-myc-IRES reporter vector *in vivo* (Evans *et al*, 2003).

Several cellular functions implicate hnRNP K as being involved in the regulation of tumorigenesis. Recently, hnRNP K was identified as a member of the p53/MDM2 pathway (Moumen *et al*, 2005). Under normal cellular conditions, hnRNP K is degraded by forming a complex with the E3 ligase MDM2. However, in cells undergoing DNA damage MDM-2 dissociates from hnRNP K through interaction with p53, resulting in increased hnRNP K expression. Furthermore, hnRNP K is a coactivator for p53 target genes and downregulation of hnRNP K in p53-positive cells, causes defects in DNA damage-induced checkpoint arrests (Moumen *et al*, 2005).

Heterogeneous ribonucleoprotein K increases the transcriptional activity of the oncogenes c-myc and c-src and translation of c-myc mRNA. A mutated version of the c-myc internal ribosomal entry site which is found prevalently in patients with multiple myeloma, is bound by hnRNP K more efficiently *in vitro* and c-myc is translated to a greater extent by hnRNP K *in vivo* (Evans *et al*, 2003). These observations suggest a possible mechanism whereby hnRNP K contributes towards multiple myeloma, by binding the mutant mRNA strand increasing translation of the oncogene c-myc (Evans *et al*, 2003). The involvement of hnRNP K in tumorigenesis is also based on the observations that growth factors regulate hnRNP K expression (Mandal *et al*, 2001) and also that hnRNP K is overexpressed in SV40-transformed cells (Dejgaard *et al*, 1994). Null and weak alleles of bancal, a *Drosophila* protein related to hnRNP K, impair adult appendage morphogenesis (Charroux *et al*, 1999; Gates and Thummel, 2000). Such a phenotype occurs owing to decreased cell proliferation in the imaginal discs showing that a homologue of hnRNP K is also necessary for cell growth.

Heterogeneous ribonucleoprotein K has also been shown to be involved in cell migration; a process necessary for cancer metastasis. Modulation of cell adhesion is an important stage of metastasis and interestingly a recently discovered structure termed the spreading initiation centre was shown to exist in the early stages of cell spreading (de Hoog *et al*, 2004). Using a lung fibroblast cell line, spreading initiation centres were shown to consist of several proteins, hnRNP K being one of them, and interfering with hnRNP K, increased cell spreading (de Hoog *et al*, 2004). It was hypothesised by the authors that hnRNP K controls cell spreading by a rate limiting process rather than inhibiting (de Hoog *et al*, 2004). Nevertheless these observations provide evidence of a connection between hnRNP K and initiation of cell spreading, a process essential for tumour cell invasion and metastasis.

Using a combination of two-dimensional (2D)-SDS-PAGE, mass spectrometry and comparing normal colon to colorectal cancer tissues, we identified hnRNP K as being overexpressed in colorectal cancer. Subsequently, the expression profile of hnRNP K with a monoclonal antibody to hnRNP K which we developed was analysed in detail using a colorectal cancer tissue microarray (TMA).

## MATERIALS AND METHODS

### Two-dimensional gel electrophoresis and peptide mass mapping

Two-dimensional gel electrophoresis and matrix-assisted laser desorption ionisation time of flight mass spectrometry (MALDI-TOF) mass spectrometry on normal colon and colorectal tissues were performed as previously described (Lawrie *et al*, 2004; Dundas *et al*, 2005). Proteins were extracted from Dukes C adenocarcinoma tissue samples and patient-matched morphologically normal colorectal mucosa (*n*=10 pairs of tumour and normal samples). Two-dimensional gel electrophoresis was performed using 3–10 pI immobilon strips. Following completion of the electrophoresis, gels were stained with Coomassie Blue to visualise proteins spots. The spots were excised from the gels and peptide mass mapping MALDI-TOF MS was performed using a PerSeptive Biosystems Voyager-DE STR mass spectrometer.

The masses of the tryptic fragments were determined using MALDI-TOF MS, and entered into the MS-Fit database-searching program (http://prospector.ucsf.edu/ucsfhtml14.0/msfit.htm). The database was restricted to human proteins but no restriction was placed on molecular weight or isoelectric point. To ensure that proteins were accurately identified a significant difference in statistical score between proteins ranked first and second in the results had to be obtained.

### Monoclonal antibody to heterogeneous ribonucleoprotein K

A monoclonal antibody to hnRNP K was produced using a method we have described previously (McFadyen *et al*, 1999). Briefly, a 10 amino-acid C-terminal peptide to hnRNP K (SVKQYSGKFF) conjugated to ovalbumin was used to immunise mice subsequently followed with a booster. The spleens of mice that demonstrated the highest antibody titres as determined by ELISA using the peptide immunogen were fused with myeloma cells. Antibody titres were assessed again by ELISA after cloning of the hybridomas. The specificity of the antibody was further confirmed by Western blotting using immortalised human cells lines (HT29 and CALU1).

### Tumour samples

This project had the permission of the Grampian Research Ethics Committee. There were 268 patients in the study and the cases were selected from the Aberdeen colorectal tumour bank. All the patients had a diagnosis of primary colorectal cancer and had undergone elective surgery for colorectal cancer, in Aberdeen, between 1994 and 2003. The tumour samples had been submitted to the Department of Pathology, University of Aberdeen for diagnosis. The tumour excision specimens had been fixed in formalin and representative blocks embedded in wax and sections stained with haematoxylin and eosin. The clinico-pathological characteristics (age, gender, site of primary tumour, degree of primary tumour differentiation and Dukes stage) of the patients included in this study are detailed in Table 1. Complete follow-up was available for all patients and ranged from 1 month to greater than 117 months. There were 75 (28%) deaths in the patient group with a median survival of greater than 117 months and a mean survival of 80 months.

### Tissue microarray

A colorectal cancer TMA was constructed as described previously (Dundas *et al*, 2005). The TMA contained primary colorectal cancer (Dukes A=53, Dukes B=104 and Dukes C=111), 111 lymph node metastasis and 53 normal morphologically colonic mucosal samples. The lymph node metastasis were from the corresponding Dukes C cases. The arrayed tumours reflected the distribution of the anatomical locations and Dukes stage of colorectal cancer in this population. A single normal colon sample was obtained from each of the 53 colorectal cancer resection specimens and each sample was acquired from at least 10 cm distant from the tumour as described previously (Dundas *et al*, 2005). One representative 1.6 mm core of tissue was taken from each donor block using a steel Menghini needle and arrayed into the recipient wax block. One section from each microarray was stained with haematoxylin and eosin to confirm the histopathological diagnosis and the adequacy of sampling. Sectioning and processing the microarray incurred a rate of core loss of 5–15% consistent with other published studies (Gulman and O'Grady, 2003).

### Immunohistochemistry

Immunohistochemistry for hnRNP K was carried out using a Dako autostainer (Dako Cytomation, Ely, UK). Sections (5 *μ*m) of the TMA were dewaxed, rehydrated and an antigen retrieval step performed. The antigen retrieval step consisted of microwaving the sections in 0.01 M citrate buffer at pH 6.0 for 20 min in an 800 W microwave oven operated at full power. The sections were then allowed to cool to room temperature. Heterogeneous ribonucleoprotein K (1 in 5) diluted in antibody diluent (Dako) was applied for 60 min at room temperature, washed with buffer (Dako) followed by peroxidase blocking 5 min (Dako) and a single 2 min buffer wash. Pre-diluted peroxidase-polymer labelled goat anti-mouse/rabbit secondary antibody (Envision™, Dako) was applied for 30 min at room temperature, followed by further washing with buffer to remove unbound antibody. Sites of peroxidase activity were then demonstrated with diaminobenzidine as the chromogen applied for three successive 5-min periods. Finally sections were washed in water, lightly counterstained with haematoxylin, dehydrated and mounted. Omitting the primary antibody from the immunohistochemical procedure and replacing it with antibody diluent acted as negative controls. For p53 staining a mouse monoclonal antibody (Dako) was used after antigen retrieval at a dilution of one in 40.

The sections were evaluated by light microscopic examination and the intensity of immunostaining in each section assessed independently by two observers (MM and GIM) using the scoring system described. There were very few discrepancies (<5% of cases) and these were resolved by simultaneous re-evaluation. Both the intensity of immunostaining and proportion of cells staining positively were assessed and the cellular compartment (nucleus v cytoplasm) in which the immunoreactivity occurred also noted. The intensity of immunostaining in each core was graded as negative=zero, weak=1, moderate=2 or strong=3 (Dundas *et al*, 2005). The proportion of cells staining positively were assessed as no cells=0, 1–25% of cells=1, 26–50%=2, 51–75%=3 and 76–100%=4. The numbers representing intensity and percentage cells stained were added together to give either a nuclear or cytoplasmic score and will now be referred to as score. The scores were then banded as follows 0=negative, 2, 3, 4=low and 5, 6, 7=high for further analysis. Tumours stained for p53 were scored as being either positive or negative.

### Statistical analysis

Statistical analyses including *χ*^2^ test, *t*-test, and Kaplan–Meier survival analysis were performed using SPSS v11.5 for Windows XP™ (SPSS UK, Ltd, Woking, UK). The log-rank test was used to determine survival differences between individual groups. We regarded *P*<0.05 as significant.

## RESULTS

### Identification of heterogeneous ribonucleoprotein K as being overexpressed in colorectal cancer

**Figure 1:**
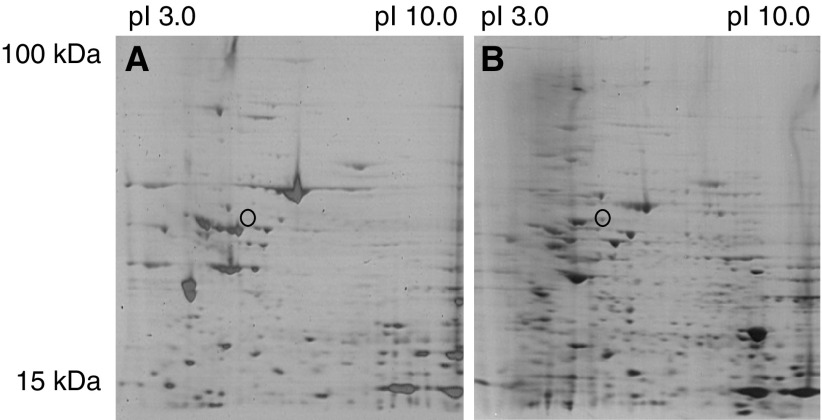
Coomassie-stained 2D SDS-PAGE gels showing whole-cell lysates of normal colon (**A**) and colorectal tumour samples (**B**). Circle highlights a protein spot present in the tumour samples but not in the normal tissue, which was identified as hnRNP K by MALDI-TOF MS analysis. Molecular weight (Mw) and pI values are shown.

To identify potential biomarkers of colorectal cancer, whole-cell lysates of normal colon and primary Duke C colorectal tumours were separated by 2D SDS-PAGE, which allowed the two protein profiles to be compared (Figure 1). Spots which were observed uniquely in the tumour samples were subsequently excised and identified by MALDI-TOF. One consistently overexpressed protein spot highlighted in tumour samples was identified using mass spectrometry, MS (Figure 1). The predicted mass of the protein and peptide mass analysis identified it as hnRNP K, the MOWSE score (20 400) was significantly higher for hnRNP K compared with the next protein.

**Figure 2:**
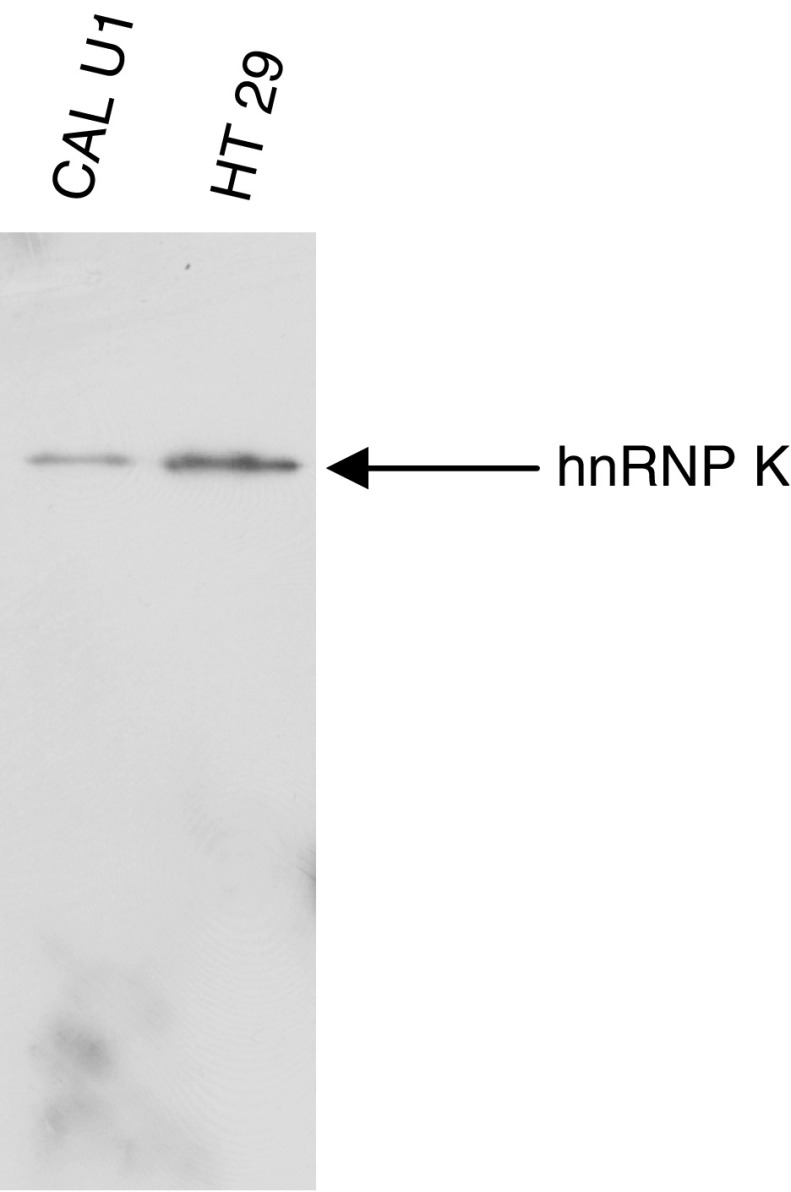
Immunoblot showing specificity of the hnRNP K monoclonal antibody. Whole-cell lysates of CAL U1 and HT 29 reveal the monoclonal antibody recognises a single band, migrating at the expected molecular weight for hnRNP K by Western blotting.

The specificity of the monoclonal antibody to hnRNP K was determined by ELISA using the immunogenic peptide (data not shown) and also by Western blotting (Figure 2) using two cells lines isolated from lung (CALU1) and colon (HT29) carcinomas. A single band migrating at the expected molecular weight was observed. The validated hnRNP K monoclonal antibody was subsequently used to immunostain the colorectal TMA.

### Cellular localisation and expression profile of heterogeneous ribonucleoprotein K in colorectal cancer

**Figure 3:**
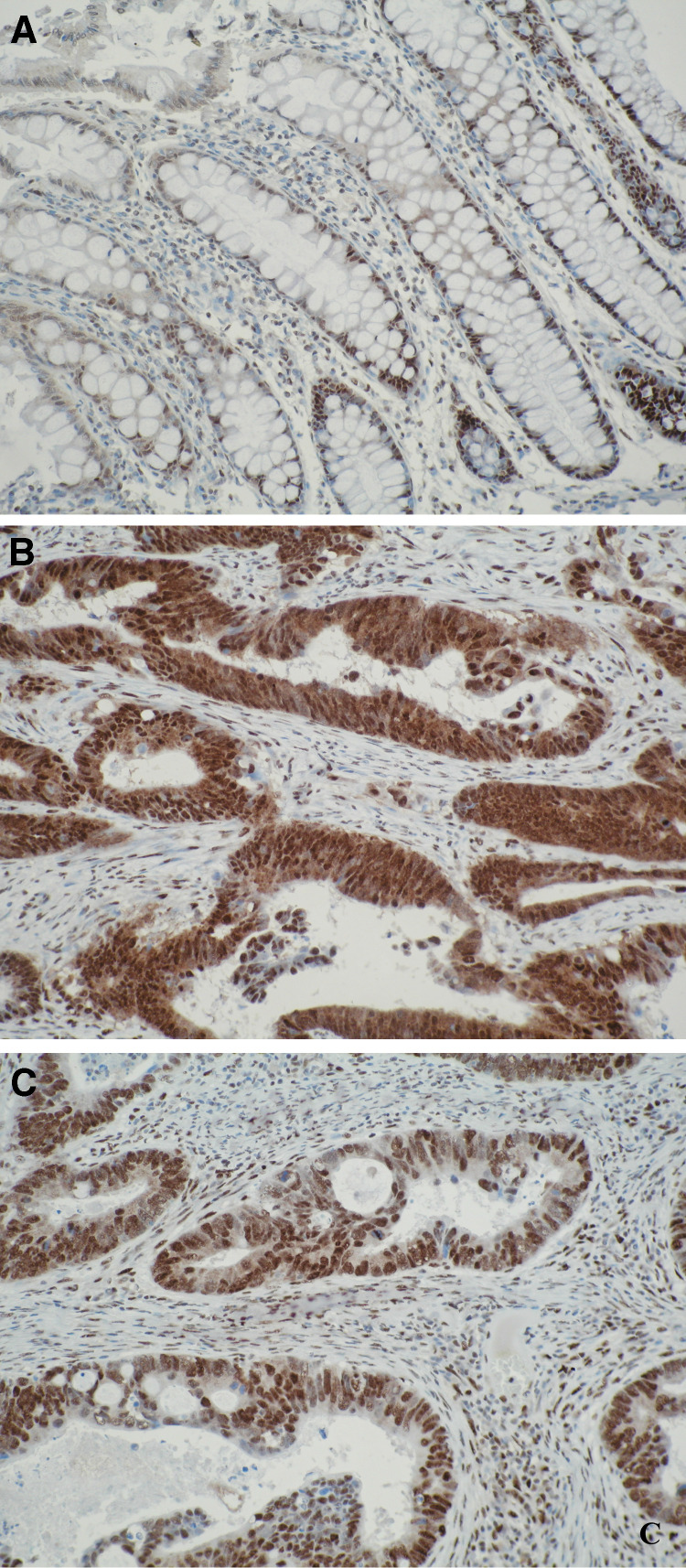
Immunoreactivity for hnRNP K in normal colon and colorectal cancer. (**A**) In normal colon, hnRNP K immunoreactivity is exclusively present in the nuclei of crypt epithelial cells. (**B**) Strong nuclear and cytoplasmic staining in primary colorectal cancer. (**C**) Strong nuclear and weak cytoplasmic staining in primary colorectal neoplasm.

In normal colon (Figure 3A) hnRNP K localisation was exclusively nuclear with no cytoplasmic expression. In primary colorectal tumour tissues however, hnRNP K localised both in the nucleus and the cytoplasm (Figure 3B and C). The same hnRNP K staining profile observed in the primary colorectal tissues was also observed in the lymph node metastasis.

**Figure 4:**
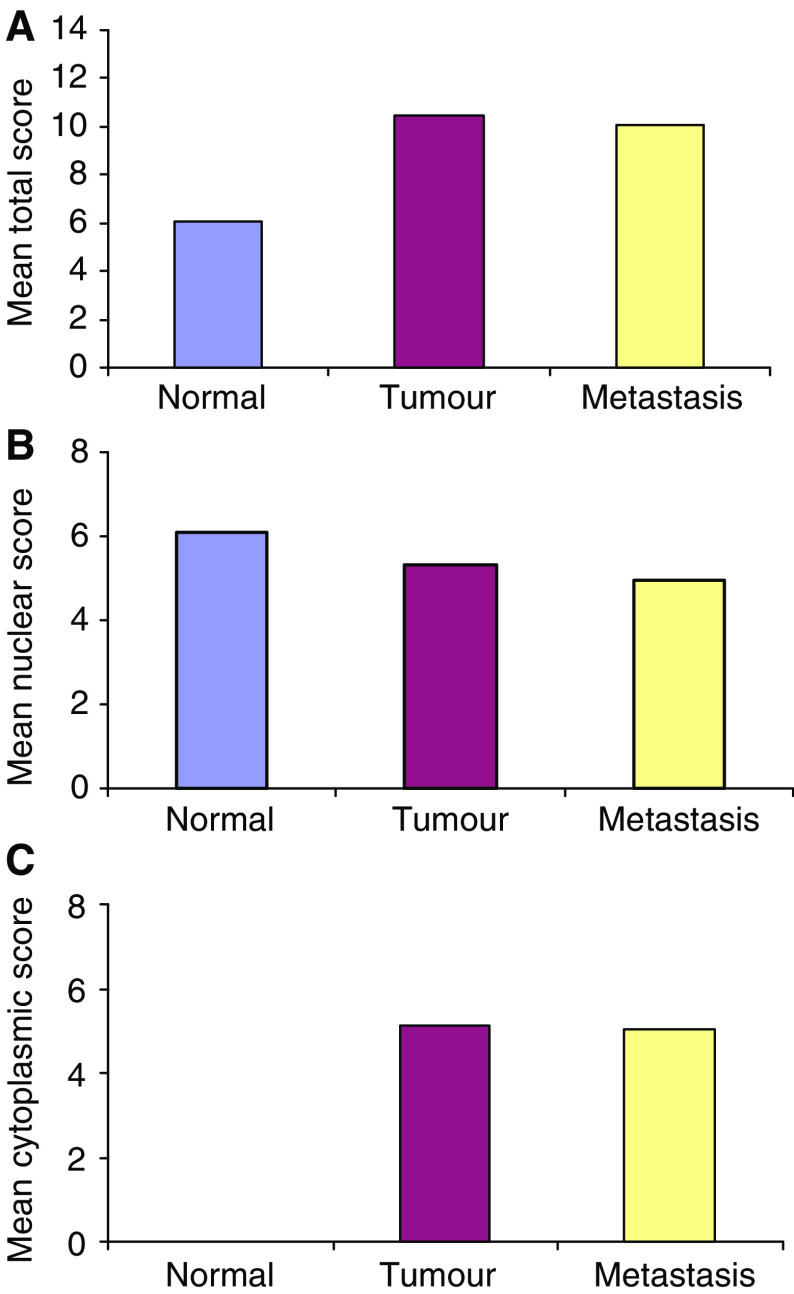
Mean score of hnRNP K in normal colon, primary tumour and metastatic tissues. (**A**) Total mean scores showed that there was a significant increase in hnRNP K expression when comparing normal colon to primary colorectal cancer (*P*<0.001). Subsequently mean scores were evaluated based on cellular localisation, nuclear (**B**) and cytoplasm (**C**). (**B**) As tumour stage progressed hnRNP K nuclear expression significantly decreased (normal to tumour *P*<0.001 and tumour to metastasis *P*=0.006). (**C**) No hnRNP K expression was observed in the cytoplasm of normal tissue but there was a significant increase in cytoplasmic immunoreactivity (*P*<0.001) in primary tumours. No difference was observed in mean hnRNP K cytoplasmic score for the tumour to metastasis transition.

In agreement with the 2D-SDS-PAGE and MALDI-TOF analysis, total mean score of hnRNP K was significantly (*P*<0.001) higher in primary tumour compared to normal colon (Figure 4A).

Mean score was subsequently analysed based on subcellular localisation. A significant decrease (comparing normal colon to primary tumour and primary tumour to lymph node metastasis *P*<0.001 and *P*=0.006, respectively) in mean nuclear stain occurred as the different stages of tumour development progressed (Figure 4B). For cytoplasmic hnRNP K mean score (Figure 4C) a significant increase was observed only when normal colon was compared with primary tumours (*P*<0.001).

### Relationship of heterogeneous ribonucleoprotein K expression, clinico-pathological parameters and survival

**Figure 5:**
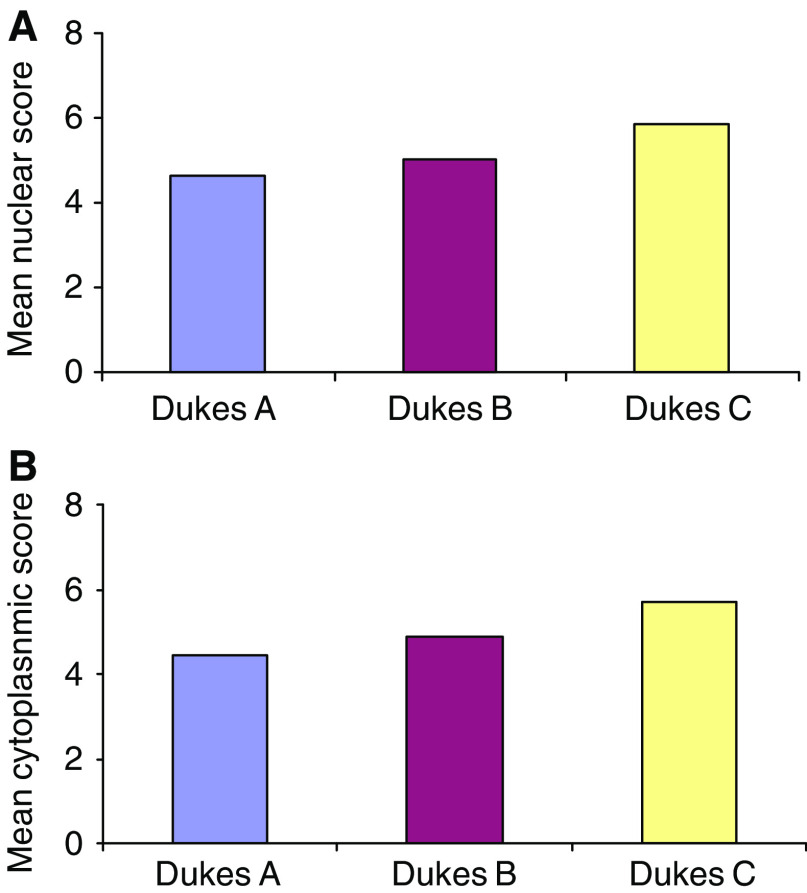
The mean nuclear and cytoplasmic score in the different stages of colorectal cancer. There are significant increases in both nuclear (**A**) and cytoplasmic (**B**) hnRNP K in Dukes C tumours (*P*=0.007 and 0.001, respectively).

There were significant increases in both nuclear (*P*=0.007) and cytoplasmic (*P*=0.001) hnRNP K in Dukes C tumours (Figure 5). There was no relationship between either nuclear score or cytoplasmic score and tumour site, tumour differentiation and gender or age.

**Figure 6:**
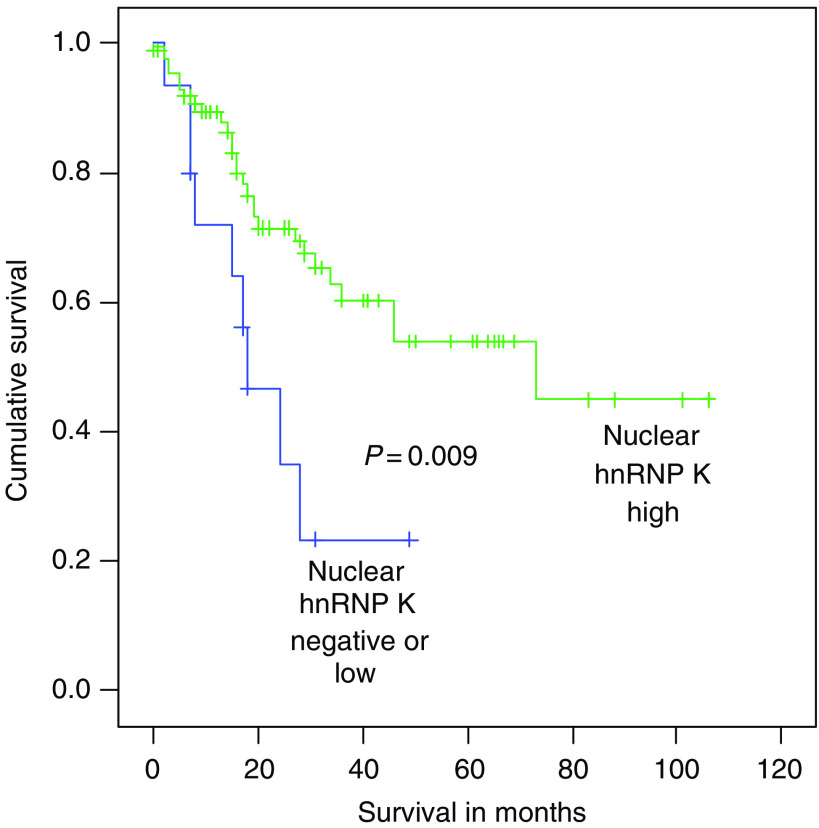
Comparison of survival in patients whose tumours with a high nuclear hnRNP K expression and those tumours with a low or moderate nuclear hnRNP K expression. There is poorer survival in those patients whose tumours with a low or moderate hnRNP K nuclear expression compared with those patients whose tumours have a high nuclear expression (log rank=6.77, *P*=0.0093).

There was no correlation between overall survival and hnRNP K nuclear or cytoplasmic expression. However, in patients with Dukes C tumours there was poorer survival in those patients whose tumours had a low or negative nuclear hnRNP K score compared with those patients whose tumour had high nuclear hnRNP K score (log rank=6.77, *P*=0.0093, Figure 6). In the poor survival group (negative or low hnRNP K nuclear expression, *n*=15) the mean survival time was 23.4 months whereas in the good survival cohort (high hnRNP K nuclear expression, *n*=87), the mean survival time was 64.1 months.

### Heterogeneous ribonucleoprotein K and p53 expression in colorectal cancer tissues

**Figure 7:**
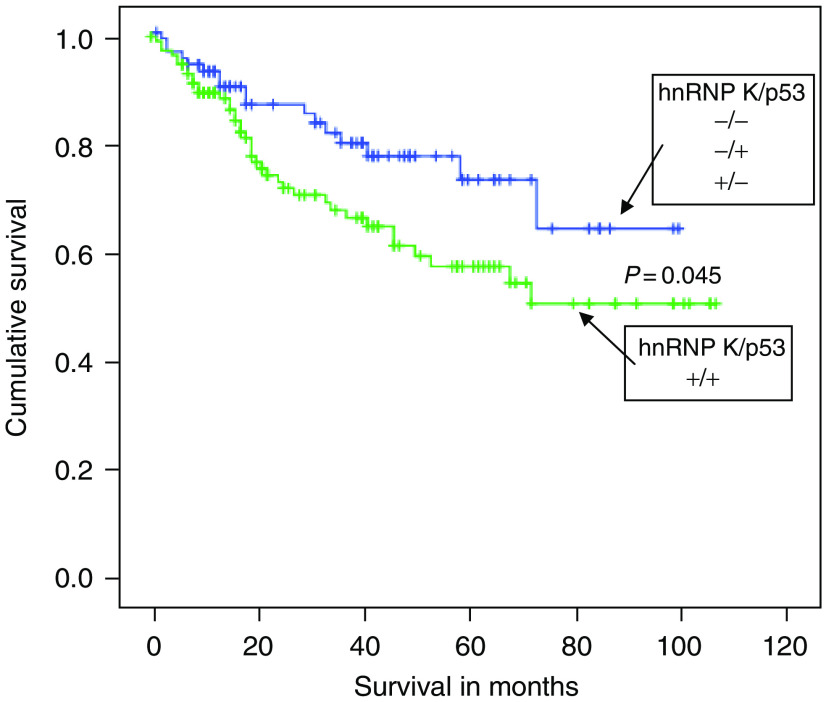
Survival analysis patients who presented tumours that were hnRNP K+/p53+had a poor survival outcome compared to patients with tumours that were either negative for both proteins or, p53+/hnRNP K− or p53−/hnRNP K+(log rank=4.001, *P*=0.045).

Heterogeneous ribonucleoprotein K and p53 expression were compared based on a scoring system of 1 and 0 for expression and absence of expression, respectively. p53 was localised exclusively in the nucleus (Table 2). The hypothesis that p53 regulates hnRNP K expression was tested first (Table 2a). Tumours were divided in to four groups p53+/hnRNP K+, p53+/hnRNP K-, p53−/hnRNP K+ and p53−/hnRNP K. There was no correlation between p53 and hnRNP K expression irrespective of hnRNP K cellular localisation. For instance, the percentage tumours which were hnRNP K nuclear or cytoplasmic+were the same irrespective of p53 expression (91.2% p53+*vs* 92.4% p53 – for hnRNP K nuclear and 93.6% p53+*vs* 92.4% p53 – for cytoplasmic hnRNP K). Heterogeneous ribonucleoprotein K also did not appear to regulate p53 expression (Table 2b), the percentage of p53+ tumours were similar irrespective of hnRNP K nuclear expression (60.9% hnRNP K nuclear+*vs* 64.7% hnRNP K nuclear −). Analysis (Figure 7) did show that there was a significant (log rank=4.001, *P*=0.045) survival trend, patients who presented p53+/hnRNP K+ colorectal tumours (*n*=119) had a poorer survival outcome compared to patients with tumours of varied p53/hnRNP K expression, *n*=85 (p53+/hnRNP K-, p53−/hnRNP K+ and p53−/hnRNP K−). The mean survival time for the good survival cohort was 77.5 months *vs* 68 months for the poor survival cohort (p53+/hnRNP K+). There were no significant survival differences within individual Dukes stages. Additionally, there were no differences between the two groups regarding patient age and gender, tumour stage, tumour site or tumour differentiation.

## DISCUSSION

Colorectal cancer is one of the commonest cancers in the Western world whose incidence is continuing to rise. A significant proportion of patients present with locally advanced disease and current therapy is based on a 5-fluorouracil regimen. Even with treatment the 5-year survival rate is still relatively poor at approximately 40%. Therefore, owing to the poor survival outcome it is important to identify biomarkers of colorectal cancer to facilitate early diagnosis. Importantly, biomarkers that are overexpressed or even more beneficial, present solely in the cancer tissues potentially act as specific targets for anti cancer therapeutics. Using a combination of 2D-SDS-PAGE, mass spectrometry and comparing normal colon to colorectal cancer tissues, we identified hnRNP K protein as being overexpressed in colorectal cancer. Further analysis using immunohistochemistry and a tissue database showed that hnRNP K was overexpressed in colorectal cancer. In support of these findings hnRNP K has been found to be upregulated in several different cancer types inclusive of lung (Pino *et al*, 2003) and liver cancer (Ostrowski and Bomsztyk, 2003). Interestingly in the cancer tissues hnRNP K localisation was aberrant; in normal colon hnRNP K was present exclusively in the nucleus whereas in tumour tissues the protein was observed both in the cytoplasm and the nucleus and in Dukes C cancers both nuclear and cytoplasmic hnRNP K was significantly increased compared with early stage tumours. Such changes in cellular localisation of hnRNP K has also been observed in smooth muscle cells (SMC). In response to serum stimulation of SMC *in vitro*, hnRNP K protein levels increased and the protein translocated from the nucleus to the cytoplasm (Laury-Kleintop *et al*, 2005). Differences in hnRNP K localisation appear to be modulated by post-translational modificiation, phosphorylation of hnRNP K by mitogen-activated protein kinase (MAPK) results in cytoplasmic localisation of hnRNP K. Serum stimulation is necessary for MAPK phosphorylation of hnRNP K which is indicative of cellular growth (Habelhah *et al*, 2001). Additionally, there is evidence to show that hnRNP K upregulation is a cause rather than an effect of proliferation; overexpression of hnRNP K in breast cancer cells significantly increases cell proliferation and growth in an anchorage-independent manner (Mandal *et al*, 2001).

Why therefore does hnRNP K accumulate in the cytoplasm of colorectal cancer cells? Heterogeneous ribonucleoprotein K has several cellular roles including transcription, mRNA splicing and translation. It is therefore tempting and not unreasonable to suggest that hnRNP K has different roles in normal colon and colorectal cancer. In normal colon cells, hnRNP K may be involved in regulating DNA process such as transcription whereas in tumour tissues hnRNP K may be involved in RNA-related functions such as shuttling of mRNAs important for cellular growth, splicing and translation. In support of this hypothesis it has already been shown that hnRNP K enhances the translation of the c-myc mRNA (Evans *et al*, 2003) and hnRNP K has also been shown to have a cytoplasmic-based role relevant to cell metastasis (Moumen *et al*, 2005).

Survival analysis showed that in Dukes C patients the stronger the nuclear hnRNP K expression the better the survival outcome. In squamous cell lung cancer high expression hnRNP B1 has also been shown to be associated with improved survival (Wu *et al*, 2003). Such observations indicate that certain hnRNP family members may be prognostic markers for colorectal cancer.

Moumen *et al* (2005) have shown that in response to DNA damage, p53 inhibits hnRNP K ubiquitin-dependent proteasomal degradation. We showed hnRNP K is overexpressed in colorectal cancer; it was therefore decided to determine whether this increase in expression was related to p53. Surprisingly, there was no correlation between p53 and hnRNP K expression. One plausible explanation is p53 may only stabilise hnRNP K expression as a consequence of DNA damage, where as during other cellular functions such as division hnRNP K expression could possibly be regulated by alternative mechanisms. In support of this reason Moumen and co-workers showed that only DNA-damaging agents such as ultra violet light or ionising radiation induced hnRNP K stabilisation through p53, whereas other stress stimuli such as hypotonic or hypertonic conditions or heat-shock failed to produce such a response. Further experiments will therefore be needed to determine how hnRNP K expression is increased and/or stabilised in colorectal cancers. The growth factors epidermal growth factor and heregulin-*β*1 have already been reported to increase expression hnRNP K mRNA and protein (Mandal *et al*, 2001) presenting two possible pathways for further analysis.

Interestingly, correlating survival with p53 and hnRNP K expression (nuclear or cytoplasmic) did show a significant trend. Patients who presented tumours that were positive for both p53 and hnRNP K expression had a poorer survival outcome compared with other combinations (p53+/hnRNP K-, p53−/hnRNP K+ and p53−/hnRNP K−). It therefore appears p53 and hnRNP K-positive tumours are more aggressive. Interestingly, p53 and hnRNP K cooperate to augment transcription of target genes (Moumen *et al*, 2005), providing one possible reason why tumours simultaneously expressing these proteins are more aggressive.

In summary, hnRNP K was shown to be overexpressed in colorectal cancer cells and its subcellular localisation was aberrant; in normal colon cells hnRNP K was exclusively nuclear whereas in tumour cells this hnRNP family member was present both in the cytoplasm and the nucleus. Additionally, Dukes C patients who presented tumours with strong hnRNP K nuclear expression had a better survival outcome.

## CONFLICT OF INTEREST

GIM is a named inventor on a patent application that The University of Aberdeen and Auvation Ltd have made to exploit the overexpression of hnRNP K in colorectal cancer as a diagnostic marker and therapeutic target.

## Figures and Tables

**Table 1:** Clinicopathological features of the microarrayed colorectal adenocarcinomas (number (%))

*Gender*	
Male	135 (50.4%)
Female	133 (49.6%)
	
*Age*	
Mean	68 years
Range	33–92 years
⩽70 years	127 (47.4%)
>70 years	141 (52.6%)
	
*Dukes stage*	
A	53 (19.8%)
B	104 (38.8%)
C	111 (41.4%)
	
*Tumour site*	
Proximal colon	95 (35.4%)
Distal colon	97 (36.2%)
Rectum	76 (28.4%)
	
*Tumour differentiation*	
Well	10 (3.7%)
Moderate	228 (85.1%)
Poor	30 (11.2%)

**Table 2:** Comparing expression of hnRNP K and p53 in colorectal cancer tissues

**(a)**		
**p53**	**hnRNP K nuclear**	**Percent of tumours**
+	+	91.2% (114/125)
+	−	8.8% (11/125)
−	+	92.4% (73/79)
−	−	7.6% (6/79)
		

**p53**	**hnRNP K cytoplasmic**	**Percent of tumours**
+	+	93.6% (117/125)
+	−	6.4% (8/125)
−	+	92.4 (73/79)
−	−	7.6 (6/79)
		

**(b)**		
**hnRNP K nuclear**	**p53**	**Percent of tumours**
+	+	60.9% (114/187)
+	−	39.1 (73/187)
−	+	64.7% (11/17)
−	−	35% (6/17)
		

**hnRNP K cytoplasmic**	**p53**	**Percent of tumours**
+	+	61.2% (117/191)
+	−	38.2% (74/191)
−	+	61.5 (8/13)
−	−	38.4 (5/13)

hnRNP K=heterogeneous ribonucleoprotein K.

The expression of p53 was compared to nuclear or cytoplasmic hnRNP K staining. Absence or presence of the protein is represented by a − or +, respectively.
